# Comparative transcriptome profiling of *Blumeria graminis* f. sp. *tritici* during compatible and incompatible interactions with sister wheat lines carrying and lacking *Pm40*

**DOI:** 10.1371/journal.pone.0198891

**Published:** 2018-07-05

**Authors:** Yuting Hu, Yinping Liang, Min Zhang, Feiquan Tan, Shengfu Zhong, Xin Li, Guoshu Gong, Xiaoli Chang, Jing Shang, Shengwen Tang, Tao Li, Peigao Luo

**Affiliations:** Provincial Key Laboratory of Plant Breeding and Genetics, College of Agronomy, Sichuan Agricultural University, Chengdu, Sichuan, China; Julius Kühn-Institut, GERMANY

## Abstract

*Blumeria graminis* f. sp. *tritici* (*Bgt*) is an obligate biotrophic fungus that causes wheat powdery mildew, which is a devastating disease in wheat. However, little is known about the pathogenesis of this fungus, and differences in the pathogenesis of the same pathogen at various resistance levels in hosts have not been determined. In the present study, leaf tissues of both *Pm40*-expressing hexaploid wheat line L658 and its *Pm40*-deficient sister line L958 were harvested at 0 (without inoculation), 6, 12, 24, 48 and 72 hours post-inoculation (hpi) with *Bgt* race 15 and then subjected to RNA sequencing (RNA-seq). In addition, we also observed changes in fungal growth morphology at the aforementioned time points. There was a high correlation between percentage of reads mapped to the *Bgt* reference genome and biomass of the fungus within the leaf tissue during the growth process. The percentage of mapped reads of *Bgt* in compatible interactions was significantly higher (at the p<0.05 level) than that of reads in incompatible interactions from 24 to 72 hpi. Further functional annotations indicated that expression levels of genes encoding H^+^-transporting ATPase, putative secreted effector proteins (PSEPs) and heat shock proteins (HSPs) were significantly up-regulated in compatible interactions compared with these levels in incompatible interactions, particularly at 72 hpi. Moreover, Kyoto Encyclopedia of Genes and Genomes (KEGG) pathway analysis suggested that genes involved in the endocytosis pathway were also enriched in compatible interactions. Overall, genes encoding H^+^-transporting ATPase, PSEPs and HSPs possibly played crucial roles in successfully establishing the pathogenesis of compatible interactions during late stages of inoculation. The study results also indicated that endocytosis is likely to play a potential role in *Bgt* in establishing compatible interactions.

## Introduction

Powdery mildew fungi (Ascomycetes, Erysiphales) infect more than 10,000 plant species [1], one of which is *Blumeria graminis* f. sp. *tritici* (*Bgt*), an obligate biotrophic fungus that grows and reproduces only on living wheat (*Triticum aestivum* L.), which is an economically important agricultural crop that is in high demand around the world [2]. Powdery mildew caused by *Bgt* has seriously reduced wheat production both in China and around the world [3–5]. To control wheat powdery mildew, 80 powdery mildew resistance (*Pm*) alleles have been identified at 53 gene loci in wheat and its relatives, most of which confer race-specific resistance [6,7]. In contrast, only one avirulence gene in *Bgt*, named *AvrPm*^*3a/f*^, is recognized by the *Pm3a* and *Pm3f* alleles and has been cloned [8], and an allele-unspecific pathogen-encoded suppressor of avirulence named *SvrPm*^*3a1/f1*^ also has been cloned [9]. Both *AvrPm*^*3a/f*^ and *SvrPm*^*3a1/f1*^ have similar expression kinetics, with a peak at haustorium formation, and only the inactive suppressor and sufficient amounts of AVR protein can active the R gene [8,10]. Similarly, in *Blumeria graminis* f. sp. *hordei* (*Bgh*), which is the other *forma specialis* of *Blumeria graminis*, only two avirulence genes, *Avra10* and *Avrk1*, have been cloned. Both genes are derived from non-LTR retrotransposons [11,12], and their limited homology at the DNA and protein level indicates that they arose from distantly related LINE families [13]. Comparatively, the pathogenesis of *Bgt* is more unclear than the resistance mechanism of wheat.

With the rapid development of sequencing technologies, whole-genome sequencing of wheat powdery mildew and barley powdery mildew has been completed, and much genomic information has become available [14,15]. Whole-genome sequencing demonstrated that the *Bgt* genome was 180 Mb and contained more than 90% transposable element (TE) sequences [15]. This result indicated that many repetitive sequences in the *Bgt* genome led to its large size, which is four times larger than that of other fungal genomes [16–18]. Genome analysis of *Bgt* showed 602 genes encoding putative effector proteins [15]. Similarly, 491 genes encoding candidate secrete effector proteins (CSEPs) have been identified in the *Bgh* genome [14]. Most *Bgt* putative effector proteins have close homologs in *Bgh*, which suggests functional conservation between the two closely related pathogens [19]. Furthermore, most candidate effector proteins share a conserved Y/F/W×C-motif in the N-terminal downstream of the signal peptides but they are highly sequence diverse, which suggests that many different targets exist in their host plants [11]. In *Bgt*, the avirulence gene *AvrPm*^*3a/f*^ and suppressor of avirulence gene *SvrPm*^*3a1/f1*^ are both typical CSEP genes [8,10]. However, in *Bgh*, the avirulence genes *Avra10* and *Avrk1* are largely distinct from CSEPs [11,12]. Although members of the CSEP family have been regarded as major determinants in pathogenesis and as good candidates for AVR proteins [10], only a few these candidate effectors have been verified functionally [20,21]. Other candidate effectors include *Bgh* effector candidates (BECs), most of which are encoded by CSEP genes [22]. Functional analysis of BECs indicated that some play a virulence role; e.g., the ribonuclease-like BEC1011 and BEC1054 (synonyms CSEP0264 and CSEP0064) are *bona fide* effectors that function within the plant cell [23]. In addition, BEC1054 may target several host proteins and thus may play a central role in barley powdery mildew virulence by acting at several levels [24]. The host-induced gene silencing of BEC1019, a metalloprotease-like protein, significantly reduces fungal colonization of barley epidermal cells, indicating that BEC1019 also plays a vital role in virulence [25].

Generally, candidate effector proteins and BECs have been suggested to be produced mostly during the formation of the haustorium, which is a specialized infection structure of *Blumeria graminis* [11, 23]. Classic studies have demonstrated that haustoria are composed of three parts: the haustorial body with filamentous lobes [26,27]; the extrahaustorial membrane (EHM), which separates the haustoria from the host cytoplasm; and the region between the EHM and haustorial body, called the extrahaustorial matrix (EHMx) [28,29]. The haustorial complex is believed to promote the uptake of carbohydrates and amino acids from a living host [26,30,18] and transfers of the effector proteins across the EHM to the host [11]. This finding indicated that powdery mildew haustoria are the most important regions for interacting with host cells, and CSEPs or BECs have been suggested to function mainly in haustoria.

In recent years, transcriptome sequencing has proven useful for gene prediction and annotation; this technique has permitted the elucidation of the temporal expression patterns of powdery mildew after infecting hosts. At the initial research of the powdery mildew pathogen transcriptome, Thomas et al. utilized expressed sequence tag (EST) analysis and serial analysis of gene expression (SAGE) to study the transcriptome during *Bgh* pathogenesis and obtained approximately 60,000 tags isolated from ungerminated conidia, germinating conidia, and appressoria of *Bgh* [31,32]. Thereafter, cDNA microarrays of *Bgh* transcript profiles during the asexual development cycle demonstrated that glycolysis and lipid metabolism pathways are involved in mature appressoria formation, epidermis infection and fungal germination. In addition, appressoria formation and function are likely regulated by the transcripts *clap1* and *cap20*, which encode a copper transporter and the CAP20 protein, respectively [33,34]. With the development of next-generation sequencing technologies, large-scale “-omics” techniques such as genomics, transcriptomics, and proteomics have been widely applied for the study of plant-powdery mildew interaction [35]. For instance, transcriptome analysis of *Golovinomyces orontii* haustoria has confirmed that transcripts involved in protein turnover, detoxification of reactive oxygen species and fungal pathogenesis are highly abundant in the haustorial EST contigs, while transcripts encoding transporter proteins for nutrient uptake are not highly abundant [36]. The comparative transcriptome of *Bgh* during early pathogenesis on barley and immunocompromised *Arabidopsis* revealed a conserved *Bgh* transcriptional program during pathogenesis compared with the natural host, barley, and genes encoding CSEPs were massively and consistently induced at high levels during haustoria formation in compatible interactions compared with incompatible interactions [37]. Previous transcriptome analysis of *Bgt* showed that candidate effector proteins genes were possibly involved in the host-pathogen interaction [15]. In a recent study, the transcriptome of *Bgt* showed that activation of the metabolism of diverse sugars, glycogen synthesis, energy production, and unsaturated fatty acid oxidation occurred during *Bgt* conidiation. A crosslink between H_2_O_2_ and Ca^2+^ signaling and involvement of some other regulators are associated with regulation of *Bgt* conidiation [38]. Proteomic studies of *Bgh* have focused on purified haustoria, haustoria in barley epidermis, conidia and secondary hyphae, and most proteins appear to be involved in metabolic pathways or biological energy production [39–42].

To determine which genes and pathways are involved in *Bgt* pathogenesis, the key stage of interaction between hosts and *Bgt* and the developmental stage of *Bgt* growth, we harvested leaf tissues at 0 (without inoculation), 6, 12, 24, 48 and 72 hours post-inoculation (hpi) with *Bgt* race 15 from hexaploid wheat lines L658 carrying *Pm40* and L958 lacking *Pm40*; these tissues were used to determine the gene expression changes in *Bgt* using RNA sequencing (RNA-seq). To determine the differences in gene expression between compatible and incompatible interactions, we focused on genes encoding H^+^-transporting ATPase, putative secreted effector proteins (PSEPs) and heat shock proteins (HSPs). In addition, we also observed and recorded the morphological growth and fungal biomass of *Bgt* in compatible and incompatible interactions at various time points after infection.

## Materials and methods

### Plant material, inoculation and tissue harvest

The susceptible hexaploid wheat line L958 without *Pm40* and the resistant sister line L658 carrying *Pm40* were selected from F_7_ population that was derived from F_3_ plants from a cross between the susceptible line MY11 and the resistant line YU25 [43], and L658 and L958 were used for studying *Bgt* differential expression during compatible and incompatible interactions. The *Bgt* fungus used in this study was a single-spore pure monoculture from *Bgt* race 15 which was collected from Wenjiang, Chengdu, Sichuan Province (latitude N30° 40’ and longitude E103° 51’) in 2011, and the virulence spectrum showed that resistance genes *Pm2*, *3b*, *3c*, *3g*, *4*, *4b*, *5a*, *5b*, *6*, *7*, *8*, *11* displayed a susceptible response to race 15, *and Pm1*, *1a*, *17*, *21*, *34*, *35*, *40*, *Pm1+2+9* remained effective. The *Bgt* fungus was maintained on susceptible wheat line CY20 through weekly transfer to new plants. L658 and L958 were cultivated in a light growth chamber (Microclima MC1750E, Snijders Scientific, Tilburg, Holland) with 16-h light/8-h dark at 18°C and 80% humidity. Seven-day-old seedlings of L658 and L958 were inoculated with shaking infected plants at densities of approximately 100–200 conidia per mm^2^. The first leaves which are the oldest leaves were collected randomly for total RNA extraction at five different time points (6, 12, 24, 48 and 72 hpi), and there were three biological replicates per time point. At the same time, samples at 0 h (without inoculation) were also harvested and were employed as the control. Next, we collected 200 mg of fresh pure conidia of *Bgt* that were cultivated for 7 days on CY20 for RNA extraction. The remaining inoculated L658 and L958 seedlings were grown for 7 days to record disease development. Five-time points for sampling were chosen based on the life cycle stages of the wheat powdery mildew pathogen [44]. All samples were immediately frozen in liquid nitrogen and stored at −80°C.

### Cytological observation of wheat leaves after *Bgt* inoculation

Three leaves were collected for each time point as described. Fungal structures within the leaves were stained using a protein-specific dye as described by Wolf and Fric [45]. The samples were then examined and photographed using bright-field microscopy (Nikon ECLIPSE 80i, Nikon Corporation, Tokyo, Japan). The histological assays were repeated three times for each time point and genotype. To determine pathogen biomass, length of germ tubes (LGT) and hyphal expansion radius (HYR) were measured using SPOT software (Diagnostic Instruments, MI, USA), and haustoria formation rates (HFRs) were manually counted.

### RNA extraction, cDNA library construction and RNA sequencing

Total RNA was extracted at each time point from inoculated leaves containing fungi using the TRIzol reagent method (Invitrogen Life Technologies, CA, USA) with three biological replicates, and *Bgt* pure conidia RNA was extracted using the total RNA Kit E.Z.N.A. (Omega Bio-tek, GA, USA) according to the manufacturer’s protocol. RNA purity and concentration were determined using a NanoDrop ND-1000 spectrophotometer (Thermo Scientific, DE, USA). RNA quantity and quality were checked using the Agilent Bioanalyzer 2100 system (Agilent Technologies, CA, USA). Only the high-quality RNA sample (OD260/280≥1.8, OD260/230≥0.5, RIN≥6.5, 28S/18S≥1.0, >3ug) was used to construct the sequencing library. One portion of RNA was used for RNA-seq, and the remaining was used for further reliable analysis.

mRNA was purified from total RNA using poly-T oligo-attached magnetic beads. Then, fragmentation was carried out using divalent cations under elevated temperature in NEBNext First-Strand Synthesis Reaction Buffer (5X). Next, cDNA libraries were constructed using the NEBNext^®^ Ultra^TM^ RNA Library Prep Kit for Illumina^®^ (NEB, USA), and library fragments of approximately 500 bp were purified with the AMPure XP system (Beckman Coulter, Beverly, USA) according to the manufacturer’s recommendations. Finally, these fragments were enriched by PCR amplification, the PCR products were purified (AMPure XP system), and library quality was assessed on an Agilent Bioanalyzer 2100 system. The library preparations were sequenced using high-throughput sequencing on an Illumina HiSeq™ 2500 platform, and 150 paired-end reads were generated. The raw data have been deposited in the NCBI sequence read archive under accession number SRP117269.

### Mapping clean reads to the reference genome and calculation of gene expression

High-quality reads were obtained by removing reads containing an adapter, reads containing poly-N and low-quality reads from raw data. These high-quality reads were then mapped to the reference genome of *Bgt* isolate 96224 [15]. Mapping was performed with TopHat2 software (version 2.0.7, Johns Hopkins University, MD, USA) [46] built on top of BOWTIE2 [47], allowing up to 2 base mismatches. Only the reads mapped to the reference genome of *Bgt* isolate 96224 were considered as *Bgt*-specific transcripts. To determine the gene expression level, the normalized expression levels FPKM (fragments per kilobase of transcript effective length per million fragments mapped to whole *Bgt* transcripts) were calculated by the Cufflinks software as previously described [48].

### Detection and functional annotation of differentially expressed genes

The *Bgt-*L658 interaction at the different time points and the *Bgt* pure conidia were considered control treatments for comparison, and the differential gene expression between the *Bgt*-L658 interaction and the *Bgt-*L958 interaction at the corresponding various time points was measured using DESeq software [49]. The false discovery rate (FDR) was calculated by adjusting the p-value using the Benjamini-Hochberg approach, and the adjusted FDR<0.05 and |log2 (fold change) |≥1.5 were set as the conditions for differentially expressed genes (DEGs).

To clarify the function of DEGs, nucleotide sequences of the DEGs were aligned against the protein database Swiss-Prot database [50] and the NCBI non-redundant “NR” database by using BLASTX, and E-value<1e-5 was set as the threshold. Putative physiological and functional categories were assigned according to Gene Ontology (GO) enrichment analysis, which was implemented using the TopGO package. To further predict metabolic pathways, KOBAS software was used to determine the statistical enrichment of DEGs in KEGG (the Kyoto Encyclopedia of Genes and Genomes) pathways [51]. GO terms and KEGG pathways with p-values<0.05 were considered significantly enriched. The DEGs were also annotated in the COG (Clusters of Orthologous Groups of proteins) database at the same time [52].

### Reliable analysis through semi-quantitative RT-PCR and real-time qRT-PCR

To further demonstrate the reliability of the RNA-seq data, 5 genes were chosen randomly from the expressed genes for semi-quantitative RT-PCR and real-time qRT-PCR experiments. Transcript one-step gDNA removal and the cDNA synthesis super mix system were used for the cDNA synthesis of *Bgt* at each time point after interacting with L658 and L958 using oligo-dT primers according to the manufacturer’s instructions. Semi-quantitative RT-PCR was performed, and each 25 μL reaction mixture conntained 1 μL of cDNA. All reactions were run under identical conditions: 4 min at 94°C for pre-degeneration; 36 cycles of 45 s at 94°C, 45 s at 60°C, and 45 s at 72°C; and a final extension of 10 min at 72°C. The primer sequences used for this detection are listed in Additional S1 Table. PCR products were visualized on 1.5% (w/v) agarose gels stained with ethidium bromide. Quantification was performed on an Eppendorf real-time qRT-PCR detection system using Stratagene SYBR Green I (TransGen Biotech, Beijing, China). The assays were performed with three technical replicates at each time point for L658 and L958 after infection with *Bgt* under the following conditions: 40 cycles of 30 s at 94°C, 5 s at 94°C and 30 s at 60°C. The melting curve was set at 60°C to 95°C with a 0.5°C increase per step. Expression values relative to the *Bgt* reference gene elongation factor 1-α (GenBank: EPQ64622.1) were calculated using the ΔΔCt method [53].

## Results

### Differences in the infection processes and fungal biomass between compatible and incompatible interactions

**Fig 1:**
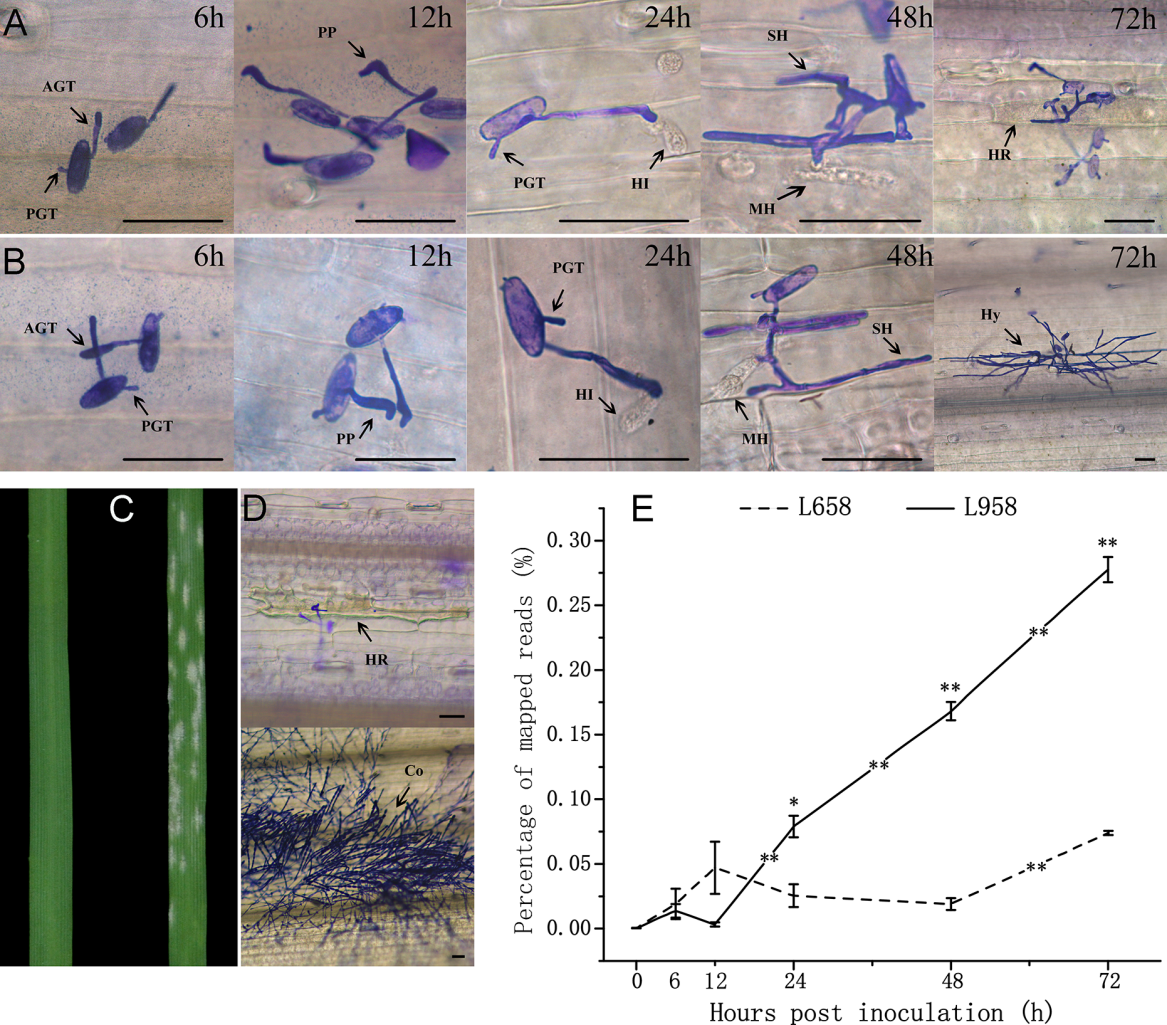
Reaction phenotypes and percentage of mapped reads of *Bgt* interacting with L658 and L958 at various time points. (A) Resistance reaction of L658 to *Bgt* at various time points. From left to right, the results are from 6, 12, 24, 48, and 72 hpi. Necrosis appeared at 72 hpi, which was the hallmark of a hypersensitive response. (B) The susceptible reaction of L958 to *Bgt* and *Bgt* hyphal growth occurred in the intercellular space at 72 hpi. (C) Samples from the left to right indicate the resistant and susceptible phenotypes of L658 and L958 leaves at 7 dpi. (D) From the top to bottom are microscopic observations of *Bgt* interacting with L658 and L958 at 7 dpi. L658 necrosis grew while numerous conidia covered the L958 leaves. AGT: appressorium germ tube; PGT, primary germ tube; PP: penetration peg; HI: initial haustoria; MH: mature haustoria; SH: secondary hyphae; Hy, hyphae; HR: hypersensitive response; Co: conidia. Dark bar indicates 50 μm. (E) The dotted and solid lines represent the rates of mapped reads from *Bgt* interacting with L658 and L958, respectively. Each result is the mean of three independent biological repeats; bars show standard deviations, and statistical significance was determined using an independent sample t-test. The asterisks represent statistically significant differences as follows: **p≤0.01, *p≤0.05. An asterisk at the top of the bars represents the difference between L658 and L958 interactions at each time point. An asterisk in the trend line represents the difference between two adjacent time points for the same genotype.

We observed pathogen growth in the early stages based on light microscopy (Fig 1A and 1B). Although *Bgt* had similar growth phenotypes and similar development times, the fungal biomass differed between the two interactions. For example, the germ tube (Fig 1A and 1B) grew rapidly in both interactions at 12 hpi, but the LGT obviously differed between these two interactions (Table 1). We also observed initial haustoria formation in both interactions at 24 hpi, but the HFRs were significantly higher in compatible interactions than in incompatible interactions (Table 1). In contrast, hyphae proliferated on the leaf surface of L958, while the resistance response was observed in numerous cells of L658 leaves at 72 hpi (Fig 1A and 1B). At the same time, the HYRs of compatible interactions were significantly higher than those of incompatible interactions (Table 1). Finally, L958 exhibited a susceptible phenotype, with numerous conidia covering the leaves, while L658 had no obvious symptoms except for a few spots of chlorosis at 7 days post-inoculation (dpi) (Fig 1C and 1D).

**Table 1:** The average biomass of *Bgt* during L658 and L958 interactions for each time point of three independent biological replications.

Target	Time interval	L658 interaction	L958 interaction	p-value
**LGT**^**a**^ **(μm)**	6 h	28.08±3.306	29.82±2.258	0.687
	12 h	46.71±0.337	37.89±2.391	0.064
**HAR**^**b**^ **(%)**	24 h	9.93±1.011	41.5±3.291	0.001
**HYR**^**c**^ **(xm)**	48 h	69.57±9.183	81.33±1.202	0.328
	72 h	120.78±29.990	261.12±18.228	0.016

^a^: The length of germ tubes, which represents the average length of primary germ tubes of total interaction sites

^b^: Haustoria formation rates, which represents the proportion of the sites where formation of haustoria in total interaction sites

^c^: Hyphal expansion radius, which represents half of the average of two perpendicular diameters of each colony.

### Transcriptome data and the percentage of mapped reads of *Bgt*

A total of 2,360,250,800 high-quality reads were obtained after a quality check and data cleaning, and few *Bgt*-specific reads were detected in the control samples of either L958 or L658 at 0 h (without inoculation) (S2 Table). In addition, we mapped clean reads of each sample to the *Bgt* reference genome. However, the percentage of reads mapped to the *Bgt* reference genome was very low in all samples, and the largest percentage of mapped reads was only 0.28% in L958 at 72 hpi (Fig 1E, S2 Table). Interestingly, although the change tendencies of the percentage of mapped reads were similar between the two interactions within 6 hpi, the percentage of mapped reads of *Bgt* on L958 was significantly higher than that of *Bgt* on L658 at 24, 48 hpi, and the difference in the percentage of mapped reads between *Bgt* on L958 and *Bgt* on L658 continuously increased with *Bgt* growth so that the largest difference in the percentage of mapped reads between them occurred at 72 hpi (Fig 1E). In addition, the change tendency in the percentage of mapped reads differed between the two interactions from 6 to 24 hpi. In *Bgt*-L658 interaction, there was an obvious increase from 6 to 12 hpi and an obvious decrease from 12 to 24 hpi. In contrast, in *Bgt*-L958 interaction, there was an obvious decrease from 6 to 12 hpi but a significant increase from 12 to 24 hpi (Fig 1E).

### Identification and annotation of DEGs

We identified 9039 transcripts, of which 6525 were annotated in the *Bgt* reference genome, and 290 of the annotated genes encoded PSEP. Further differential expression analysis of these transcripts showed that no DEGs were found in *Bgt* between the L658 and L958 interactions at 6 and 24 hpi. We also found only one DEG at both 12 and 48 hpi, encoding a plasma membrane H^+^-ATPase and a mannosidase GPI-anchored membrane protein, respectively (Table 2). We identified 260 DEGs for *Bgt* between the L658 and L958 samples at 72 hpi, including 224 annotated genes, and 208 (92.9%) were up-regulated in the L958 interaction compared with the L658 interaction at 72 hpi (Table 2), of which 60 DEGs expressed specifically in L958 interaction (S3 Table). Similarly, 177 DEGs, including 120 annotated genes were found between the L958 interaction at 72 hpi and the *Bgt* conidia at 7 dpi (Table 2), of which 10 DEGs expressed specifically in *Bgt* conidia (S3 Table).

**Table 2:** DEG numbers and annotated DEGs.

L958 vs. L658^a^	All DEGs	Annotated DEGs	Up-regulated	Down-regulated
**6h**	0	0	0	0
**12h**	1	1	0	1
**24h**	0	0	0	0
**48h**	1	1	0	1
**72h**	260	224	208 (92.9%^c^)	16
**S72 vs. *Bgt***^**b**^	177	120	11	109 (90.8%^d^)

Numbers indicate the number of genes differentially expressed (FDR<0.05, |log_2_ fold change |≥1.5), which were compared in pairs at each time point. Significant expression differences occurred almost exclusively at 72 hpi.

^a^: *Bgt* interaction with L958 (compatible interaction) compared with *Bgt* interaction with L658 (incompatible interaction) at various time

^b^: *Bgt* interaction with L958 at 72 hpi compared with *Bgt* conidia at 7 dpi

^c^: Proportion of up-regulated DEGs with annotated DEGs

^d^: Proportion of down-regulated DEGs with annotated DEGs.

**Fig 2:**
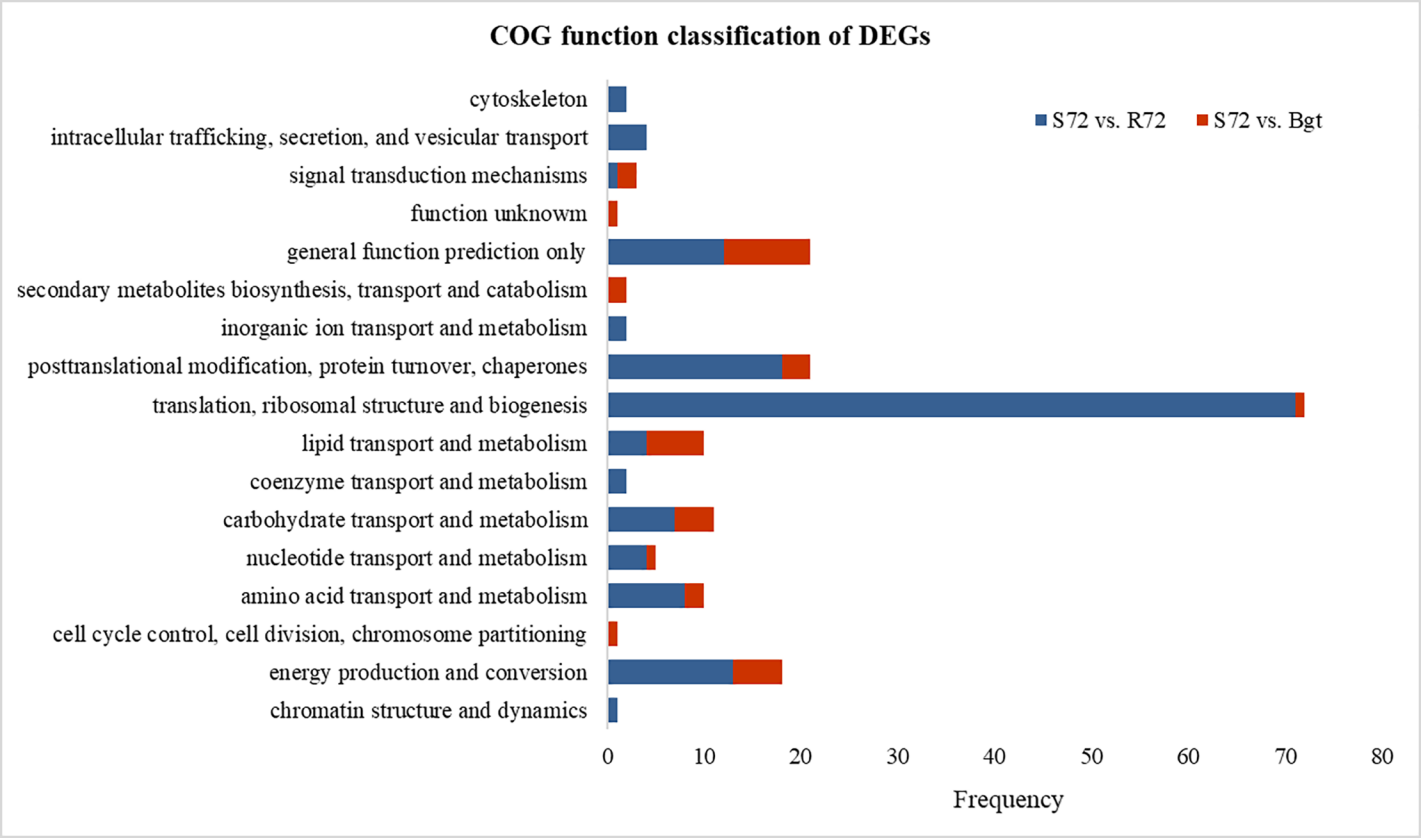
COG function classifications of DEGs in groups from L958 compared with those from L658 and *Bgt*. S72 vs. R72 represents *Bgt* interaction with L958 compared to *Bgt* interaction with L658 at 72 hpi; S72 vs. *Bgt* represents *Bgt* interaction with L958 at 72 hpi compared with *Bgt* conidia at 7 dpi.

Further COG analysis was performed with the DEGs of L958 interaction compared with L658 interaction at 72 hpi, and the result showed that 71 (31.7%) DEGs belonged to translation, ribosomal structure and biogenesis; 18 (8.0%) belonged to posttranslational modification, protein turnover and chaperones; 13 (5.8%) belonged to energy production and conversion; 8 (3.6%) belonged to amino acid transport and metabolism; and 7 (3.1%) belonged to carbohydrate transport and metabolism (Fig 2). The overwhelming proportion of elements associated with and protein synthesis and turnover suggest that proteins abundant in compatible interaction at 72 hpi. The major metabolic pathways of DEGs in the L958 interaction at 72 hpi compared with those in *Bgt* conidia at 7 dpi were similar despite the lower numbers of DEGs (Fig 2). In addition, we found four different metabolic pathways for DEGs in the L958 interaction compared with those in the L658 interaction at 72 hpi: cytoskeleton; coenzyme transport and metabolism; intracellular trafficking, secretion and vesicular transport; and chromatin structure and dynamics. These metabolic pathways were absent from the DEGs in the L958 interaction at 72 hpi compared with those in *Bgt* conidia at 7 dpi (Fig 2).

**Fig 3:**
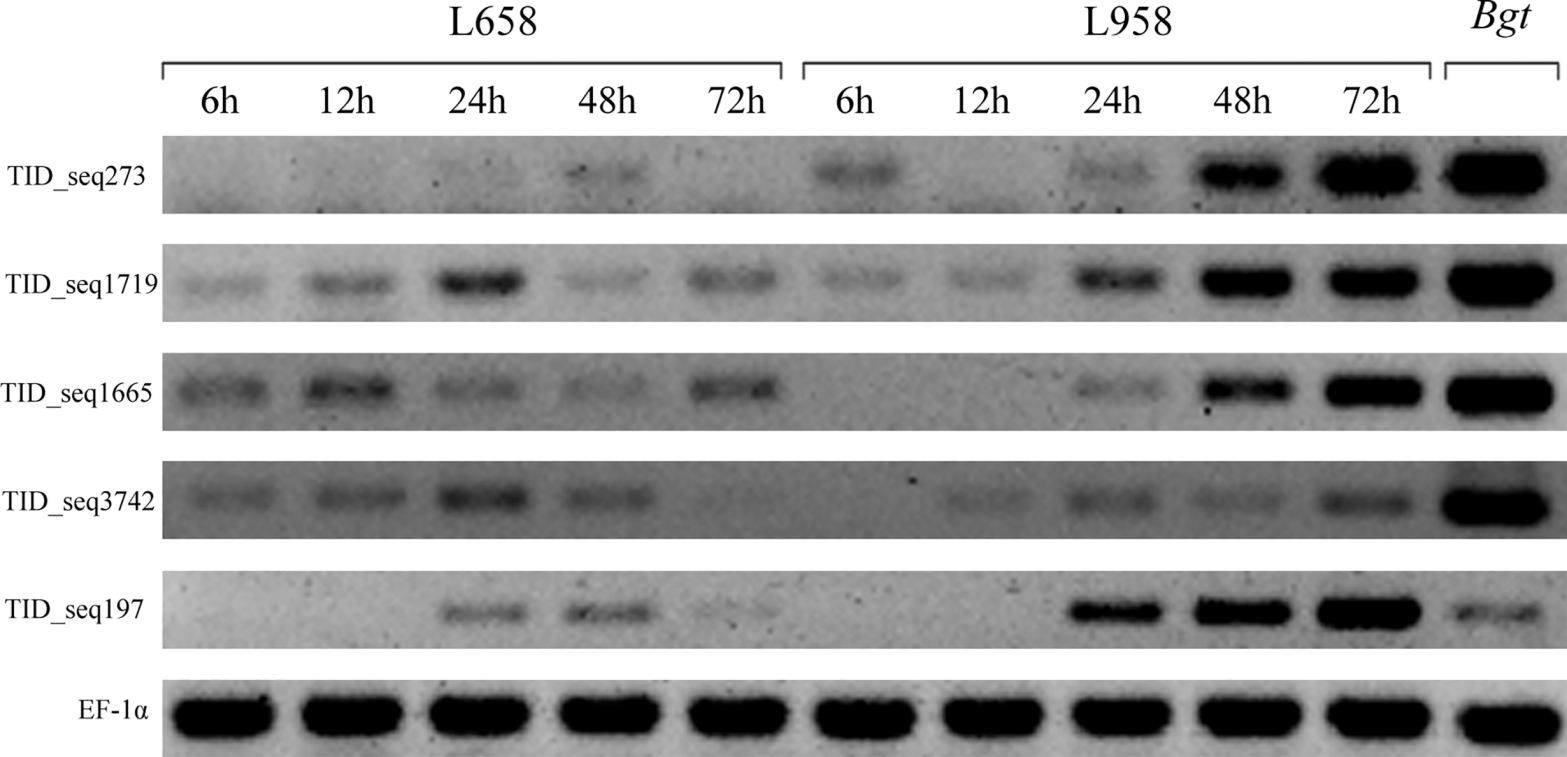
Semi-quantitative RT-PCR analyses of the L658 interaction, L958 interaction and *Bgt* conidia of the five target genes and one reference gene at each time point. Five DEGs (TID_seq273, TID_seq1719, TID_seq197, TID_seq1665, TID_seq3742) were randomly chosen. Elongation factor 1-alpha (EF-1α) was chosen as the reference gene.

**Fig 4:**
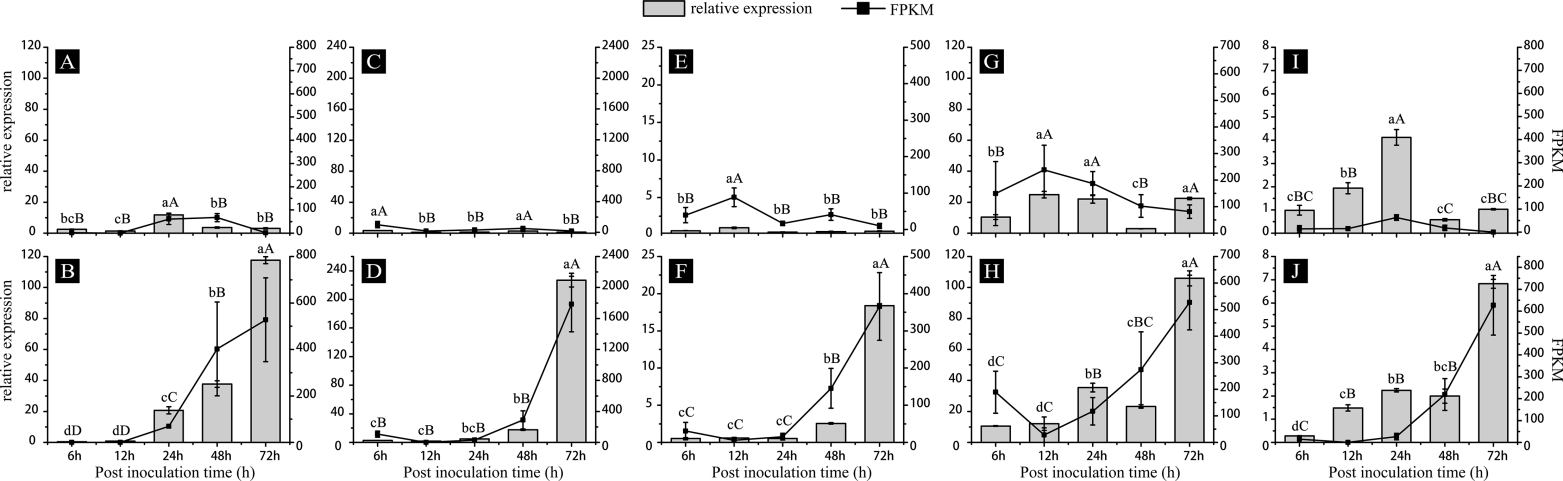
qRT-PCR of cDNA from a time-course of L958 interactions and L658 interactions of five target genes. (A), (C), (E), (G), and (I) represent TID_seq197, TID_seq273, TID_seq1665, TID_seq1719, and TID_seq3742 in L658 interactions, respectively; (B), (D), (F), (H), and (J) represent TID_seq197, TID_seq273, TID_seq1665, TID_seq1719, and TID_seq3742 in L958 interactions, respectively. Elongation factor 1-alpha (EF-1α) was used as a reference gene. The bar chart represents the relative expression levels validated by qRT-PCR, and the line chart represents the FPKM of RNA-seq. Different letters indicate statistically significant difference in relative expression of each gene among different time-points. The capital letter represents p < 0.01 while lowercase represents p < 0.05. Analyses were performed with SPSS Statistics 19.0.

To demonstrate the reliability, five randomly chosen DEGs (TID_seq1719, TID_seq197, TID_seq273, TID_seq1665, TID_seq3742) were employed to execute both semi-quantitative RT-PCR and qRT-PCR. Semi-quantitative RT-PCR showed that there was no expression in the uninfected leaves of the L958 and L658 interactions at 0 h (S1 Fig) and that the highest expression in *Bgt* conidia occurred at 7 dpi, except for the expression of TID_seq197 (Fig 3). In addition, the differential expression of these five genes was detected in both interactions at all time points after infection (Fig 3). The relative expression of these genes in the L958 and L658 interactions was highly correlated with RNA-Seq FPKM values (S4 Table, Fig 4). All relationships between the relative expression values of the 5 DEGs detected by qRT-PCR and RNA-Seq FPKM were significant at p = 0.05; most relationships were significant at p = 0.01 in the compatible interaction (S4 Table). We also found that some relationships were not significant at p = 0.05 in the incompatible interaction (S4 Table), mainly due to the low expression of this gene as well as the large detected error in both qRT-PCR and RNA-seq (Fig 4).

### Identification of pathogenic proteins and interesting DEGs

We identified 8 DEGs encoding PSEPs between the L658 and L958 interactions at 72 hpi, of which 7 genes were up-regulated and only one down-regulated in the L958 interaction compared with the L658 interaction (Table 3). In addition, 4 out of the 8 DEGs carried the conserved N-terminal Y/F/W×C motif (Table 3). Similarly, there were also 7 DEGs encoding PSEPs between the L958 interaction at 72 hpi and the *Bgt* conidia at 7 dpi; of these DEGs, 6 were up-regulated and only one down-regulated in the L958 interaction compared with the *Bgt* conidia at 7 dpi (Table 3). Additionally, 4 out of the 7 DEGs carried the conserved N-terminal Y/F/W×C motif (Table 3). Further analysis found that both the gene down-regulated in the L958 interaction compared with the L658 interaction at 72 hpi (TID_seq6419) and that down-regulated in the L958 interaction at 72 hpi compared with the *Bgt* conidia at 7 dpi (TID_seq1719) lacked the conserved N-terminal Y/F/W×C motif (Table 3).

**Table 3:** Differentially expressed three types genes annotated in NR, KEGG and Swiss-port database.

Transcript ID		Accession No. in NCBI	Putative function	KEGG_pathway	S72 vs. R72^d^	S72 vs. *Bgt*^*e*^	Motif^f^	Length of amino acid^g^
**PSEP (NR**^**a**^**)**	TID_seq148	EPQ67823.1	putative secreted effector protein	—	up (4.15)	up (6.10)	—	206
	TID_seq197	EPQ67773.1	putative secreted effector protein	—	up (4.23)	up (5.61)	YxC	263
	TID_seq2629	EPQ65328.1	putative secreted effector protein	—	up (lnf)	up (7.76)	—	143
	TID_seq6013	EPQ61929.1	putative secreted effector protein	—	up (lnf)	up (12.07)	YxC	110
	TID_seq1173	EPQ66816.1	putative secreted effector protein	—	up (lnf)	—	FxC	130
	TID_seq1300	EPQ66693.1	putative secreted effector protein	—	up (5.50)	—	—	168
	TID_seq4971	EPQ63054.1	putative secreted effector protein	—	up (lnf)	—	YxC	110
	TID_seq6419	EPQ61518.1	putative secreted effector protein	—	down (-3.23)	—	—	260
	TID_seq1719	EPQ66269.1	putative secreted effector protein	—	—	down (-2.75)	—	155
	TID_seq2965	EPQ65025.1	putative secreted effector protein	—	—	up (8.64)	YxC	111
	TID_seq4396	EPQ63571.1	putative secreted effector protein	—	—	up (10.50)	YxC	137
**H**^**+**^**-ATPases (KEGG**^**b**^**)**	TID_seq1665	EPQ66305.1	V-type H^+^-transporting ATPase 16 kDa proteolipid subunit	Oxidative phosphorylation; Phagosome	up (2.90)	—	—	146
	TID_seq5553	EPQ62470.1	V-type H^+^-transporting ATPase subunit A	Oxidative phosphorylation; Phagosome	up (4.21)	—	—	609
	TID_seq2055	EPQ65941.1	F-type H^+^-transporting ATPase subunit alpha	Oxidative phosphorylation	up (3.49)	—	—	558
	TID_seq3347	EPQ64576.1	F-type H^+^-transporting ATPase subunit beta	Oxidative phosphorylation	up (4.19)	—	—	382
	TID_seq349	EPQ67625.1	F-type H^+^-transporting ATPase subunit c	Oxidative phosphorylation	up (3.62)	—	—	157
	TID seq 6498	EPQ61480.1	H^+^-transporting ATPase	Oxidative phosphorylation	up (2.02)	—	—	976
**HSPs (Swiss-port**^**c**^**)**	TID_seq273	EPQ67700.1	Heat shock 70 kDa protein 2	Spliceosome; Protein processing in endoplasmic reticulum; Endocytosis	up (4.63)	—	—	648
	TID_seq1603	EPQ66372.1	Heat shock protein 90	Protein processing in endoplasmic reticulum; Plant-pathogen interaction	up (4.59)	—	—	701
	TID_seq2143	EPQ65860.1	Heat shock protein 78	—	up (lnf)	—	—	802
	TID_seq2513	EPQ65459.1	Heat shock protein hsp88	—	up (4.83)	—	—	709
	TID_seq2705	EPQ65318.1	10 kDa heat shock protein	—	up (lnf)	—	—	80
	TID_seq3365	EPQ64594.1	Heat shock protein 60	RNA degradation	up (4.28)	—	—	577
	TID_seq4183	EPQ63849.1	30 kDa heat shock protein	Protein processing in endoplasmic reticulum	up (lnf)	—	—	207
	TID_seq643	EPQ67331.1	Heat shock protein sti1 homolog	—	up (6.16)	—	—	559
	TID_seq787	EPQ67191.1	Heat shock protein sks2	—	up (5.49)	—	—	66
	TID_seq113	EPQ67869.1	30 kDa heat shock protein	Protein processing in endoplasmic reticulum	—	down (-2.10)	—	205

^a^: Putative protein function predicted in NR database

^b^: Putative protein function predicted in KEGG database

^c^: Putative protein function predicted in Swiss-port database

^d^: The regulation of DEG in *Bgt* interaction with L958 compared with *Bgt* interaction with L658 at 72 hpi, and the numbers in parentheses represents Log_2_ fold change. “lnf” represents the Log_2_ fold change is infinity which means the DEG expressed specifically in L958 interaction at 72hpi

^e^: The regulation of DEG in *Bgt* interaction with L958 at 72 hpi compared with *Bgt* conidia at 7 dpi, and the numbers in parentheses represents Log_2_ fold change

^f^: The type of the conserved Y/F/W×C-motif in the N-terminal of predicted proteins

^g^: The length of predicted proteins (amino acids)

We also identified genes encoding H^+^-transporting ATPase as important and interesting DEGs for differentiating compatibility and incompatibility between pathogen and host. One down-regulated gene and six up-regulated genes encoding H^+^-transporting ATPase exhibited differential expression in the L958 interaction compared with the L658 interaction at 12 and 72 hpi, respectively (S2 Fig).

Finally, we identified 9 DEGs encoding HSPs between the L958 and L658 interactions at 72 hpi; all nine genes were up-regulated in the L958 interaction compared with the L658 interaction (Table 3). In addition, we also found another one DEG, which were different from the above 9 DEGs encoding HSPs between the L958 interaction at 72 hpi and the *Bgt* conidia at 7 dpi. Moreover, the expression levels of the gene were down-regulated in the L958 interaction at 72 hpi compared with the *Bgt* conidia at 7 dpi (Table 3).

### Identification of important pathway-related compatibility and incompatibility

KEGG analysis showed that four DEGs were involved in the endocytosis pathway (S5 Table), and all four genes were up-regulated in the L958 interaction compared with the L658 interaction at 72 hpi (S5 Table). Among these four DEGs, TID_seq1665 and TID_seq273 encoded H^+^-transporting ATPase and HSP70, respectively. We did not detect differences in expression between the L958 interaction at 72 hpi and the *Bgt* conidia at 7 dpi.

## Discussion

Although several studies have investigated powdery mildew pathogens in both barley and *Arabidopsis* [33,34,24,20], fewer studies have investigated the *Bgt* pathogen in wheat [15, 38]. In present study, the different transcript expression patterns of *Bgt* during compatible and incompatible interactions and the relationships with fungal morphological growth have been determined.

In our study, the percentage of mapped reads of *Bgt* was very low in the two interactions at all time points (S2 Table). Previous studies also showed that the percentage of mapped reads of *Bgt* was low (only 0.6% in a mixed sample of samples harvested at 4, 8, 12, 24 and 48 hpi) [15]. This is a common phenomenon in the early gene expression profiles of other obligate biotrophic fungi [37,15], and in our study, it could be caused by the large hexaploid wheat transcript pool of wheat and small amount of *Bgt* biomass in the mixed samples (Tables 1 and S2).

Previous studies have demonstrated that changes in fungal morphology are highly associated with the temporal and spatial expression profiles of fungal transcripts [54,55]. In our study, the differential morphological growth in the two interactions was accompanied by large differences in the changes in the percentage of mapped reads. For example, the morphological differences in *Bgt* between compatible interaction and interactions were detected early at 12 hpi (Table 1), whereas different change tendencies of the percentage of mapped reads were detected at 6 hpi (Fig 1E). This result suggested that gene expression influencing the establishment of *Bgt* could occur within 12 hpi, and different gene expression changes from 6 hpi to 12 hpi could play crucial roles in regulating the establishment of compatible and incompatible interactions. In addition, the dramatic growth differences of *Bgt* at 72 hpi agreed well with the significantly differential expression at the transcriptome level between compatible and incompatible interactions (Fig 1A, 1B and 1E; Tables 1 and 2). Overall, comparing the changes between morphological growth and gene expression, we found that the differences in *Bgt* morphological growth were accompanied by differences in gene expression at the transcriptome level.

To determine which DEGs were interesting, we focused first on the genes encoding PSEPs. Previous studies have shown that in the *Bgt* genome, 602 putative effector genes (which account for 9.2% the total gene complement) may be involved in host-pathogen interactions [15], and most up-regulated DEGs in *Bgh* in compatible interactions compared with incompatible interactions at 24 hpi encoded CSEPs [37]. In our study, 290 genes which account for 4.4% the annotated genes encoding PSEPs. Moreover, 8 and 7 DEGs encoding PSEPs were identified between the L958 or L658 interaction at 72 hpi and between the L958 interaction at 72 hpi and the *Bgt* conidia at 7 dpi, respectively (Table 3). In addition, more than 87% of the genes up-regulated at 72 hpi in the L958 interaction compared with the *Bgt* conidia at 7 dpi encoded PSEPs (Table 3); this result implies that PSEPs play important roles in *Bgt* pathogenicity.

Previous studies also confirmed that genes of pathogens causing powdery mildew encoding secreted effectors have commonly induced expression profiles in various species [37,15]. Despite only a small proportion of these candidate effectors being verified functionally, some studies have demonstrated that secret effectors could contribute to powdery mildew virulence in early infection [37,24,20], and some secret effectors could also interact with host pathogenesis-related proteins or proteins related to defense and response to pathogens in the host, thereby weakening the resistance of the host to pathogens [56,24,15,21]. We further found that the genes encoding PSEPs were expressed only after infection, especially from 24 to 72 hpi (formation of the haustoria and hyphae), and most of these genes were not expressed initially in early infection (Figs 3, 4 and S2). Therefore, this result indicated that the genes encoding PSEPs were usually expressed non-constitutively and were expressed only by induction after infecting the host. In addition, *Bgh* candidate effectors expressed at different stages of barley infection have different functional roles [37,20,21]. In this study, we found that PSEP gene expression increased gradually after *Bgt* inoculation and peaked from 48 to 72 hpi (S2 Fig). At the same time, semi-quantitative RT-PCR and qRT-PCR results showed that genes encoding PSEPs had different profiles of temporal expression (Figs 3 and 4). Some genes, such as TID_seq1719, were induced very quickly in the early stages, usually within 24 hpi (Figs 3, 4 and S2), while other genes, such as TID_seq197, were induced in the late stages, usually at 48 hpi (Figs 3, 4 and S2). From this evidence, we could suggest that some PSEPs expressed in the early stage after pathogen infection are possible virulence factors that enhance fungal pathogenicity, that the other PSEPs expressed in later stages are more likely used to combat proteins related to defense and response to pathogens in the host and that genes encoding both kinds of PSEPs are usually up-regulated at various stages after infection and are favorable for the establishment of compatibility.

Generally, among *Bgh* CSEPs are two major types of effector families: one comprising short proteins (100–150 amino acids) with a high expression level in haustoria and one consisting of longer proteins (300–400 amino acids) with low levels in haustoria but with higher levels during penetration [22]. Most genes encoding secreted proteins are up-regulated during haustoria formation in *Bgh* [57]. Similarly, in our study, most PSEPs up-regulated at 72 hpi comprised short proteins (100–170 amino acids) (Table 3, Figs 3, 4 and S2). However, we found that one gene (TID_seq6419) encoding PSEP with long proteins (260 amino acids) was down-regulated in the L958 interaction compared with the L658 interaction at 72 hpi (Table 3) but had high expression levels at 6 hpi in the L958 interaction and at 12 hpi in the L658 interaction (S2 Fig). In addition, previous reports showed that most CSEPs have a conserved N-terminal Y/F/W×C motif [22]. In this study, most secrete effector proteins also had this motif, and all were up-regulated in compatible interactions. However, two PSEPs lacking the Y/F/W×C motif (TID_seq6419 and TID_seq1719) were down-regulated in compatible interactions (Table 3). This implied that *Bgt* PSEPs with different amino acid lengths and the presence or absence of Y/F/W×C motif possibly have different expression profiles at transcript levels similar to those found in *Bgh* and may have different functions at different infection stages.

Second, we would like to investigate to the genes encoding H^+^-ATPase because it is a popular view that plasma membrane H^+^-ATPase plays an important role in the interaction between pathogens and hosts [58,59]. In fact, various studies have found that genes encoding H^+^-transporting ATPase are up-regulated in *Bgh* and *Magnaporthe oryzae* during appressoria formation [34,60]. A specific proton pump in which H^+^-ATPase is highly involved is also abundant in *Colletotrichum higginsianum* during appressoria formation after infection [61]. In our study, the expression of DEGs encoding H^+^-transporting ATPase peaked at 6 hpi (formation of the appressoria) (S2 Fig). In addition, previous studies also showed that the expression of H^+^-ATPase is high during haustoria formation and decreases during the formation of penetration pegs [34,61,62]. We also found one down-regulated gene and five up-regulated genes encoding H^+^-transporting ATPase in the L958 interaction compared with L658 interaction at 12 hpi (formation of the penetration pegs) and 72 hpi (formation of the hyphae), respectively (Table 3). COG annotation showed that 15 DEGs were involved in the transport and metabolism of both amino acids and carbohydrates at 72 hpi (Fig 2), for which H^+^-ATPase may be needed for this process [27,18]. The differential regulation of H^+^-transporting ATPases at the transcriptional level at different developmental stages possibly responds to different requirements [61,63,64]. Overall, it is reasonable to assume that H^+^-transporting ATPase of *Bgt* may influence the formation of appressoria and penetration pegs for invading the host at early infection stages and driving the uptake of nutrition at later infection stages.

HSPs are also interesting for determining the mechanism of compatibility interactions. In *Bgh*, HSPs have been identified as the main proteins associated with fungal pathogenicity [37]. In our study, transcripts encoding HSPs were also massively induced at 72 hpi in the L958 interaction (Table 3, S2 Fig). Previous reports have indicated that the HSPs of *Candida albicans*, *Aspergillus fumigatus*, and *Ustilago maydis* are essential for intracellular homeostasis, morphogenesis, growth and stress adaptation, all of which are related to fungal pathogenicity and virulence [65,66]. Similarly, it is implied that some HSPs can enhance the capacity of pathogens to attack and that others can reduce the capacity of host defenses in *Bgt*.

We found that DEGs involved in endocytosis were up-regulated in compatible interactions compared with incompatible interactions after *Bgt* infection (S5 Table). Endocytosis is essential for the acquisition or removal of macromolecules and particles from the extracellular medium and for controlling intercellular communication, signal transduction, and cellular and organismal homeostasis in eukaryotic cells [67]. In previous studies, endocytosis internalized pathogenicity factors, such as toxic proteins, into wheat [68] and mediated the uptake of ferrichrome in *Fusarium graminearum* [69]. In addition, *Bgh* multivesicular body (MVB)-mediated endocytosis acted as an intermediate stage in the delivery of fungal virulence factors to the host cell and potentially activated elicitor receptors from the plant surface [70,71]. In our study, four DEGs were involved in the endocytosis pathway in the compatible interactions (S5 Table); the cellular components in which these DEGs function are the extracellular region, fungal-type vacuoles and actin filament bundles, which are mainly responsible for vesicle-mediated transport (S5 Table). This result indicated the possibility of exchange between the haustoria and the plant via endocytosis.

## Conclusions

The percentage of reads mapped to the *Bgt* reference genome was very low for all time points of compatible and incompatible interactions, but there were high correlations with fungal biomass. These results potentially suggest that the differences in morphological growth were accompanied by differences in gene expression at the transcriptome level. At early infection stages, H^+^-transporting ATPase may act as the key for entering hosts, which involves first breaking through the host defenses in compatible interactions while functioning similarly to incompatible interactions without the key to enter the host. After *Bgt* entered the host, it formed haustoria. The high expression of PSEPs and HSPs may indicate that most possibly acted as virulence factors to enhance pathogenicity at this stage. However, the insufficient accumulation of PSEPs and HSPs in incompatible interactions resulted in a weak attack on the host. At later infection stages, PSEPs and HSPs were possibly used to suppress the host defense response. In addition, H^+^-transporting ATPase may be involved in nutrient uptake at later infection stages in compatible interaction. Overall, H^+^-transporting ATPase, PSEPs and HSPs may play a vital role in successfully establishing pathogenesis among compatible interactions. Furthermore, endocytosis is likely an important pathway for exchanging substances as the pathogen interacts with the host.

## Supporting information

S1 Fig Semi-quantitative RT-PCR analyses of the five target genes and one reference gene at 0 h (without inoculation). (TIF)

S2 Fig The heatmap illustrates the differential expression of *Bgt* genes encoding H^+^-transporting ATPase, PSEPs and HSPs of L958 interactions and L658 interactions at various timepoints. The differential expression patterns were based on the normalized FPKM, green represents low expression level, and red represents high expression level.
(TIF)

S1 Table List of forward and reverse primers designed to amplify *Blumeria graminis* f.sp.*tritici* during infection on wheat leaves. (XLSX)

S2 Table Number of RNA-Seq reads mapped to the wheat reference genome or the *Blumeria graminis* f.sp.*tritici* isolate 96224 reference genome. (XLSX)

S3 Table The DEGs expressed specifically in L958 interaction and *Bgt* conidia. (XLSX)

S4 Table The correlation between relative expression (qRT-PCR) and FPKM (RNA-seq) of five DEGs. (XLSX)

S5 Table Function annotation of DEGs involved in endocytosis in the L958 interaction compared with the L658 interaction at 72 hpi. (XLSX)
