# Supplementary figures and images for: Comparative transcriptome profiling of Blumeria graminis f. sp. tritici during compatible and incompatible interactions with sister wheat lines carrying and lacking Pm40
Source: PLoS One. 2018 Jul 5;13(7):e0198891. doi: 10.1371/journal.pone.0198891 (PMC6033381; doi:10.1371/journal.pone.0198891)

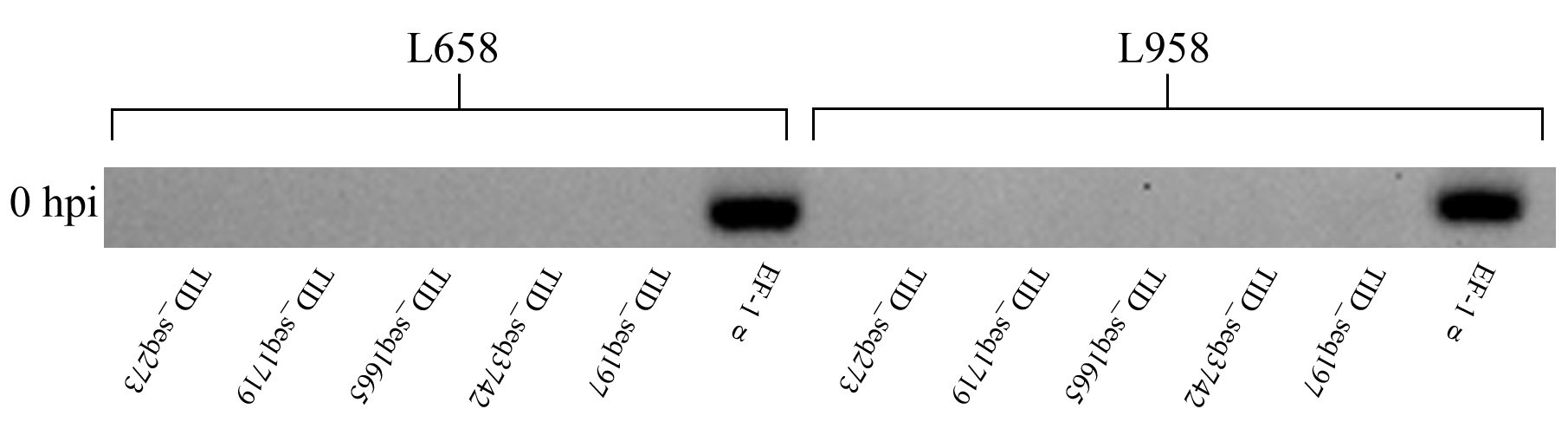

Supplement: S1 Fig — (TIF) [file pone.0198891.s001.tif]

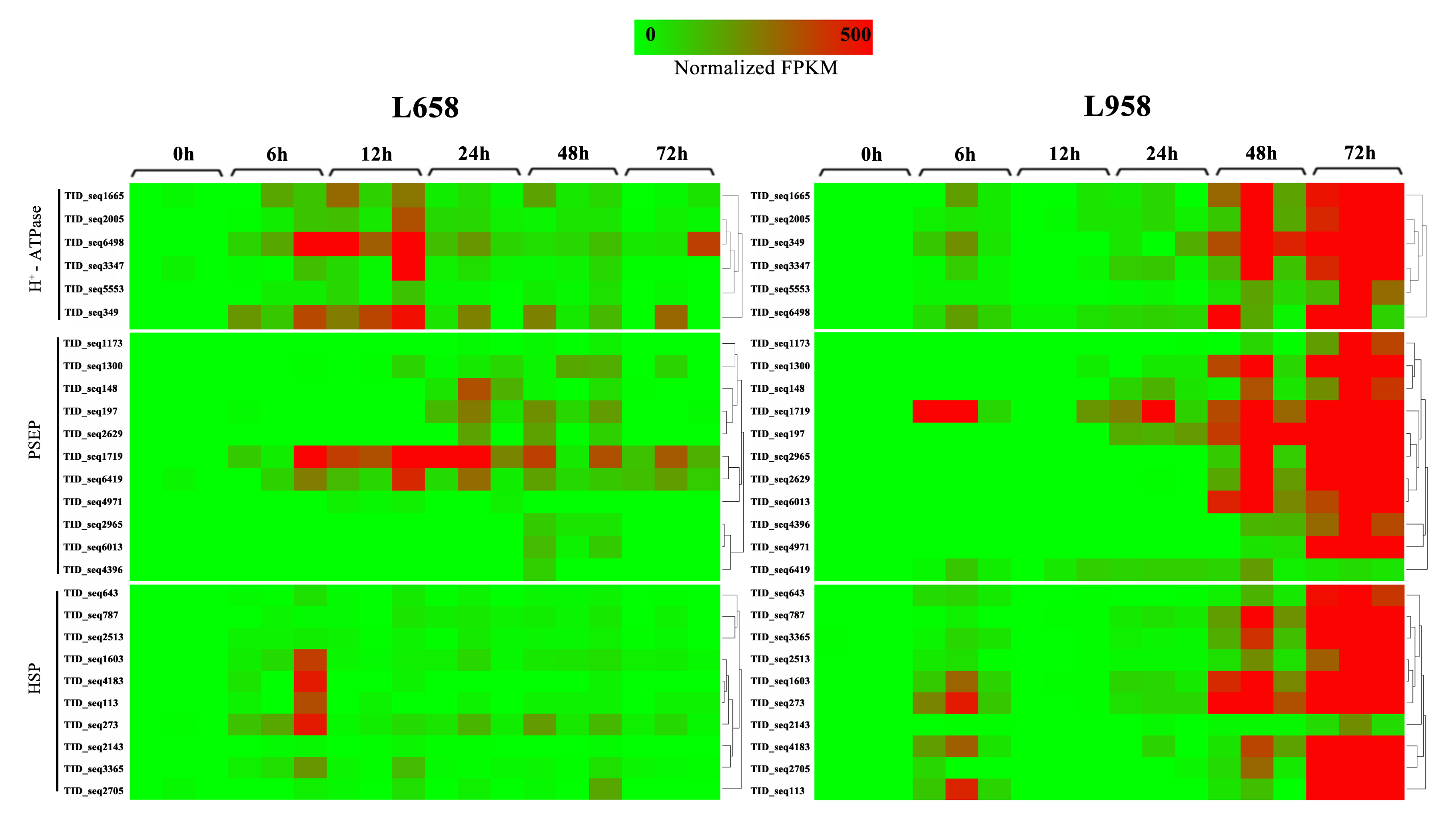

Supplement: S2 Fig — The differential expression patterns were based on the normalized FPKM, green represents low expression level, and red represents high expression level. (TIF) [file pone.0198891.s002.tif]
